# 

**Published:** 1899-02-11

**Authors:** 


					THE HOSPITAL. "The standard of highest purity,"?Lancet, Flb. Hth, 1899.
CADBURY'S vet IIT fe* CADBURY'S
cocoa MM JE^ cocoa
^=J I^j_? NO DRUGS OR ALKALIES USED.
? ? __ ?  ?    DRW1CH HOSfil-TAK
\.saair> WcBTcnM luFiauitr
No. 646. Vol. XXV. lSatttbday,
Feb. IIih, 1899,
-n/on nu^rzi-JAJL * ?
?] PB.IOE TWOPENCE.

				

